# Food Allergies and Eating Disorders: An Exploration of Screening Prevalence and Associated Factors Among Adults

**DOI:** 10.3390/nu18172890

**Published:** 2026-09-03

**Authors:** Jessica Hine, Kirsty Andresen, Rose-Marie Satherley, Christina J. Jones

**Affiliations:** 1 School of Psychology, University of Surrey, Guildford GU2 7XH, UK; 2 Surrey & Borders Partnership NHS Trust, Leatherhead KT22 7AD, UK; 3 Department of Non-Communicable Disease Epidemiology, London School of Hygiene & Tropical Medicine, London WC1E 7HT, UK

**Keywords:** food allergy, eating disorders, disordered eating, screening, adults, prevalence

## Abstract

**Background/Objectives**: Food allergy (FA) affects up to 10% of adults and requires ongoing dietary vigilance that may increase vulnerability to eating disorders (EDs), yet evidence in adults remains limited. This study compared ED screening and history between adults with and without food allergy (FA), and explored factors associated with positive screening among those with FA. **Methods**: This cross-sectional study recruited 371 adults (134 with FA, 237 without) via an online survey. Positive screening required meeting thresholds on both the Eating Attitudes Test-8 (EAT-8) and the Sick, Control, One, Fat, Food (SCOFF); ED history and treatment were assessed via self-report. Group differences were examined using chi-square tests and, for the primary comparison, age- and sex-adjusted logistic regression. Among those with FA, correlations identified candidate predictors of positive screening, which were entered into a further logistic regression. **Results**: FA status was not associated with positive screening for an ED (37.3% vs. 37.1%; *p* = 0.972), including after adjusting for age and sex. FA was significantly associated with a greater lifetime history of EDs (36.4% vs. 20.7%; *p* = 0.001) and ED treatment (17.9% vs. 10.1%; *p* = 0.032). Among those with FA, positive screening was independently predicted by ED history and shorter allergy duration in a multivariable model (Nagelkerke R^2^ = 0.14); however, allergy duration and age were highly correlated, so this effect could not be shown to be independent of age. **Conclusions**: FA alone was not statistically associated with current positive screening but was linked to lifetime history and treatment, suggesting that this relationship may be more evident earlier in the FA experience. ED history and shorter allergy duration were associated with positive screening among those with FA, though the duration effect may partly reflect age; sex was linked to screening only at the bivariate level, given too few male positive cases to model. Findings are preliminary, given the FA-unvalidated screening tools used, warranting longitudinal validated research.

## 1. Introduction

Food allergy (FA) is an adverse immune response triggered by exposure to a specific allergen via ingestion, inhalation, or skin contact [1]), affecting up to 10% of adults [2,3]). In the absence of a cure, management relies on elimination diets and rescue medication [4], and this psychosocial burden may increase vulnerability to disordered eating [5]. Eating disorders (EDs) are severe mental illnesses characterised by persistent, disrupted eating behaviours, including restriction, binge eating, and compensatory behaviours, which significantly impair functioning and carry the highest mortality rate of any psychiatric illness [6], with a 3.39 times increased risk of death relative to the general population [7]). Adults are particularly vulnerable to mortality due to the cumulative physical effects of prolonged illness [8]. Chronic diseases requiring strict dietary adherence are increasingly linked to elevated ED risk [9]. Managing food intake, adapting one’s lifestyle around food, and monitoring bodily sensations for a possible reaction can heighten this vulnerability [10,11,12].

FA severity predicts psychological distress [13], quality of life impairment [14,15], and hypervigilance around allergens which can progress into rigid, excessive restriction and disordered eating, particularly where professional guidance is lacking and avoidance extends beyond medical necessity [16,17]. This can foster obsessional thinking about food and reinforce the belief that restriction and monitoring are necessary for safety, with relief from anxiety further strengthening avoidance behaviour [12,18]. Social eating, central to daily life, is often experienced as a source of anxiety and loss of spontaneity due to meticulous planning and fear of negative social evaluation, contributing to loneliness and reinforcing the association between food and threat [16,19,20,21,22,23]. Positive feedback or health improvements following restriction may unintentionally reinforce disordered patterns.

Most research in this area has focused on children, adolescents, and their carers [11]. Findings indicate that children with FA are more likely to be underweight and to show abnormal eating behaviours [23] and symptoms of anorexia nervosa [24] when compared with the general population, with early food-induced anaphylaxis associated with later ED risk [10] and 40% showing symptoms of avoidant/restrictive food intake disorder (ARFID) [25]. Evidence in adults remains comparatively limited but suggests that two-thirds experience psychological distress and poor health-related quality of life [26], with elevated anxiety, depression, and food-related fear linked to EDs and barriers to recovery [27,28]. A systematic review found widely varying ED prevalence estimates (0.8–62.9%) among adults with FA [29], alongside reports of excessively limited diets, inadequate calorie consumption, and rigid, restrictive relationships with food [11,20,30,31]. Compared with other chronic conditions, the association between FA and EDs appears to be stronger, suggesting that FA may play a distinct mediating role in disordered eating [11], with adults also showing elevated food neophobia and ARFID symptoms linked to psychological distress and fear of a reaction [32].

Given that half of all FA develops in adulthood [2], the limited evidence base in adult populations represents a significant gap. The current study was designed to extend the findings of a paediatric cohort study conducted in Poland by Wróblewska et al. [33] to an adult population. This study was selected in order to use a comparable case-classification approach for ED status (rather than only screening-tool cut-offs) and reported the findings across a graded range of allergy severity/management demands, offering a directly extendable framework despite the age difference in samples in a strand of the literature that otherwise contains very few comparable FA–ED studies to draw on. This study aimed to (1) compare the proportion of adults with and without FA who screened positive for an ED and who reported a lifetime ED history, and (2) explore the clinical and demographic factors associated with positive screening among adults with FA. It was hypothesised that adults with FA would show higher rates of positive screening for ED than adults without FA (Hypothesis 1), and that specific clinical and demographic factors would be independently associated with positive screening among those with FA (Hypothesis 2).

## 2. Materials and Methods

### 2.1. Design

This was a cross-sectional study, with data collected from each participant at a single time point via an online questionnaire. This design enabled examination of the proportion of self-reported positive screening for an ED amongst those with and without FAs, alongside relevant demographic and clinical covariates. The study methodology was selected with consideration for the available assessments, participant population, and timeframe.

### 2.2. Participants and Recruitment

Participants were eligible if they were (i) fluent in English and (ii) aged 18 or above, and they were asked to disclose whether any FA was medically diagnosed or self-reported. Recruitment was opportunistic, via poster distribution, word of mouth, and social media, with an optional prize draw and university participation credit offered as incentives. Data were anonymised; identifying details for the prize draw and consent forms were collected and stored separately from the survey responses to maximise confidentiality. A sensitivity power analysis indicated that the achieved sample (N = 371) provided 80% power, at α = 0.05, to detect effects as small as w = 0.15 (Cohen’s w) for the primary chi-square comparisons, and effects as small as w = 0.25 for analyses restricted to the FA subgroup (n = 128–134). No a priori power calculation was conducted, as recruitment was opportunistic and time-limited; this sensitivity analysis is reported to contextualise the precision of the study’s null and significant findings. Ethical approval was obtained from the University of Surrey Ethics Committee prior to data collection.

### 2.3. Measures

Data were collected using an online survey administered via Qualtrics. The survey covered demographic information and clinical characteristics related to FA (e.g., length of FA diagnosis in years, allergen type and number, adrenaline auto-injector prescription). Sex assigned at birth was recorded as a binary variable (male/female). Gender was recorded separately as a three-category variable (man/woman/non-binary). Ethnicity was recorded using five categories (White; Asian or Asian British; Black, Black British, Caribbean or African; Mixed or multiple ethnic groups; Other), consistent with standard UK census categories. The survey also included two validated self-report measures assessing positive screening for an ED: the EAT-8 and the SCOFF. Validated self-report measures were used to assess positive screening for an ED as per the previous study [33]: meeting the cut off screening scores for the EAT-8 [34] and SCOFF [35] concurrently, rather than either measure alone. A score of 2 or more for males or 3 or more for females on the EAT-8, and a score of 2 or more on the SCOFF, indicated a positive screen [33]. Sex-specific EAT-8 cut-offs (≥2 for males, ≥3 for females) are recommended because item endorsement patterns and normative score distributions differ by sex, and applying a single threshold would misclassify a disproportionate number of men or women. The SCOFF shows good sensitivity (0.80) and specificity (0.93) for detecting ED cases and is highly recommended for screening [36], while the EAT-8 is a valid and reliable measure with good internal consistency and test–retest reliability, suitable for efficient screening in large general population samples [34,37]. In the present study, the EAT-8 demonstrated good internal consistency in both the non-FA (α = 0.82) and FA (α = 0.84) groups; the SCOFF showed poorer internal consistency in both groups (α = 0.54 and 0.62, respectively). ED history and treatment were captured via two binary self-report items only (ever/never; treated/not treated) and no diagnostic sub-type, including ARFID, was recorded. For those reporting a history of EDs, a further question was asked to determine whether this was current (defined as a self-reported ongoing ED at the time of survey completion) or resolved (defined as a self-reported past ED with no current symptoms).

### 2.4. Procedure

Participants accessed the survey link and were presented with a participant information sheet, including details of ED or FA support services (repeated at survey end) and encouragement to consult their general practitioner if concerned about their eating habits, followed by an online consent form; both were available to download. Participants completed the demographic questions, FA-relevant questions where applicable (e.g., length of FA diagnosis in years, allergens, treatment), the SCOFF and EAT-8, and finally questions about their ED history and treatment. Participants could then opt to be redirected to a separate questionnaire to enter the prize draw. The survey took approximately 10–15 min to complete.

### 2.5. Data Analysis

Data were analysed using IBM SPSS Statistics, Version 31.0 [38]. Descriptive statistics summarised the demographic and clinical characteristics, and analyses were organised by hypothesis. Hypothesis 1 (adults with FA have a higher proportion screening positive for an ED than adults without FA) was tested using chi-square tests of independence across four variables: positive screening (EAT-8/SCOFF cut-off, present/absent), lifetime ED history (ever/never), receipt of ED treatment (yes/no), and, among those reporting a lifetime history, current versus resolved status. This combined approach compared proportions based on validated screening measures alongside the participants’ own diagnostic and treatment history, providing a fuller picture of ED burden across groups.

To explore clinical and demographic factors associated with positive screening among individuals with FA, Pearson’s correlations were conducted with pairwise deletion; this is mathematically equivalent to the phi coefficient for binary–binary pairs and to point–biserial correlation for continuous–binary pairs (age, length of FA in years), both computed via the standard Pearson procedure in SPSS. For the purposes of the correlation analysis, ethnicity was recoded as a binary variable (white vs. non-White), given the small cell sizes in several ethnicity categories. Variables significantly associated with positive screening were considered for entry into a multivariable logistic regression. Where two candidate predictors were highly correlated (r > 0.60), the more clinically relevant variable was retained to avoid multicollinearity. On this basis, length of FA was retained over age, as it more directly reflects cumulative exposure to dietary restriction and FA management as the mechanism of theoretical interest. Odds ratios (OR) with 95% confidence intervals (CI) were reported to aid interpretation. Statistical assumptions were assessed prior to analyses, and significance was set at *p* < 0.05.

## 3. Results

### 3.1. Demographics and Clinical Characteristics

In total, 382 participants began the questionnaire, and 11 entries were removed from analysis due to incompleteness, resulting in a final sample of 371 participants. Of these, 134 (36.1%) reported a FA and 237 (63.9%) did not. Of the 134, 90 reported a medically diagnosed FA and 44 reported a self-reported FA. A three-group sensitivity analysis (medically diagnosed FA vs. self-reported FA vs. no FA) for the primary screening outcome was run: 38.9% of the medically diagnosed group, 34.1% of the self-reported group, and 37.1% of the no-FA group screened positive, with no significant difference across the three groups (χ^2^(2, N = 371) = 0.29, *p* = 0.864, Cramér’s V = 0.03). Furthermore, each pair of groups were examined directly: no FA vs. medically diagnosed (χ^2^(1, N = 327) = 0.09, *p* = 0.769, φ = 0.02), no FA vs. self-reported (χ^2^(1, N = 281) = 0.15, *p* = 0.701, φ = 0.02), and medically diagnosed vs. self-reported (χ^2^(1, N = 134) = 0.29, *p* = 0.590, φ = 0.05). Although the absence of a significant difference between medically diagnosed and self-reported FA does not establish equivalence between these groups, the consistently similar screening rates across all pairwise comparisons supported combining them for the primary analysis in order to retain statistical power. In total, 319 participants were born female (86%) and 52 were born male (14%). Of these, 315 (84.9%) identified as women, 50 (13.5%) as men, and 6 (1.6%) as non-binary. Of these, 282 identified as white (76.4%); 51 as Asian/British Asian (13.8%); 23 as mixed/multiple ethnic groups (6.2%); 8 as Black, Black British, Caribbean, or African (2%); and 5 as other (1.3%) (Table 1).

Among those with FA, the most commonly reported allergens were tree nuts (49, 36.6%), peanuts (45, 33.6%), cow’s milk (22, 16.4%), cereals containing gluten (20, 14.9%), eggs (18, 13.4%), and crustaceans (14, 10.4%). Fruit-related allergies (e.g., kiwi, apple, stone fruits) were also common (26, 19.4%), alongside oral allergy/pollen–food syndrome presentations (12, 9.0%) (which occurs when the immune system cross-reacts between proteins in pollen and similar proteins found in food, typically producing mild, localised oral or throat symptoms rather than the more severe systemic reactions seen in classic FA). Length of FA ranged from 1–64 years (18.92 ± 12.37). In terms of clinical management, adrenaline auto-injector ownership was reported by the majority of the medically diagnosed group (58, 63.7%) compared with a small minority of the self-diagnosed group (2, 4.7%).

### 3.2. Proportion Screening Positive and Treatment of EDs

Contrary to expectations, FA status was not associated with the likelihood of screening positive for an ED χ^2^(1, N = 371) = 0.001, *p* = 0.972, φ = 0.002, with 37.3% of those with FA and 37.1% of those without FA meeting a positive screening result. After adjustment for age and sex, the association remained non-significant in a multivariable logistic regression (N = 371, adjusted OR = 1.26, 95% CI [0.79, 2.01], *p* = 0.336). Considered separately, 62.3% of participants screened positive on the EAT-8 alone and 40.2% on the SCOFF alone; the combined definition used throughout this manuscript, requiring both cut-offs to be met, is therefore more conservative than either instrument individually. A significant association was found between FA status and ever having had an ED χ^2^(1, N = 369) = 10.77, *p* = 0.001, φ = 0.17. Participants with FA were more likely to report a history of an ED (36.4%) compared with participants without FA (20.7%). A further chi-square test was conducted, restricted to the subset of participants who reported a lifetime history of an ED (n = 91), to examine whether FA status was associated with current vs. resolved ED status within this group. The association was not significant, χ^2^(1, N = 91) = 1.68, *p* = 0.195, φ = 0.14, although a greater proportion of participants with FA reported a current ED (48.8%) compared with those without FA (35.4%). A chi-square test indicated a significant association between FA status and receiving treatment for an ED χ^2^(1, N = 371) = 4.60, *p* = 0.032, φ = 0.11. Those with FA were more likely to report previous ED treatment (17.9%) than those without FA (10.1%).

### 3.3. Clinical and Demographic Characteristics Associated with EDs

Missing data across the candidate predictor variables were minimal (< 5%); at the item level, two participants had missing data on ED history and four on length of FA, accounting for the reduced sample sizes reported. Amongst those with FA, positive screening for an ED was significantly correlated with length of FA (r = −0.21), ED history (r = 0.29), sex (r = 0.26), and age (r = −0.19, all *p* < 0.05), such that screening was more likely among those with a shorter FA duration, a prior ED history, female sex, and younger age (Table 2). Of note, sex was used in analyses because sex-specific cut-off scores are recommended for the EAT-8. Treatment for ED showed a non-significant positive association (r = 0.16, *p* = 0.060). No significant correlations emerged with adrenaline auto-injector prescription, number of allergens, or ethnicity. Among predictor variables, treatment for ED was strongly correlated with ED history (r = 0.62), and length of FA with age (r = 0.74), raising multicollinearity concerns; adrenaline auto-injector prescription and number of allergens were moderately correlated (r = 0.46; Table 2).

A multivariable logistic regression examined the independent contribution of ED history and length of FA (years) to positive screening for an ED. Age and treatment for ED were excluded due to strong correlations with length of FA (r = 0.71) and ED history (r = 0.62), respectively, to avoid multicollinearity. As only 1 in 18 male participants (5.6%; compared with 49 of 116 female participants (42.2%)) screened positive for an ED, sex could not be included in the multivariable logistic regression. The overall model was significant (χ^2^(2) = 13.87, *p* < 0.001) and fit the data well (Hosmer–Lemeshow *p* > 0.05), correctly classifying 68.8% of cases and explaining 10.3–14% of variance (Cox and Snell/Nagelkerke R^2^). Length of FA was a significant predictor of positive screening for an ED, with longer FA duration associated with lower odds of screening positive for an ED (B = −0.035, Wald χ^2^(1) = 4.39, *p* = 0.036, OR = 0.97, 95% CI [0.94, 1.00]). A sensitivity analysis substituting age for FA duration produced a near-identical effect (B = −0.038, *p* = 0.038), and entering both together rendered neither significant (*p* = 0.445 and *p* = 0.378, respectively), confirming that duration and age cannot be statistically disentangled in this sample. Having a history of EDs was also a significant predictor, with individuals reporting a prior history of disordered eating having over three times greater odds of screening positive for an ED compared with those with no such history (B = 1.120, Wald χ^2^(1) = 8.17, *p* = 0.004, OR = 3.07, 95% CI [1.42, 6.61]; Table 3).

## 4. Discussion

This study found that there was no statistically significant association between FA and positive screening for an ED in adults, even when adjusting for age and sex. The observed bivariate effect size was small (φ = 0.002), and the sample was adequately powered to detect an effect of w = 0.15 or larger; however, the CI around the adjusted estimate remains compatible with a potentially meaningful association, and these findings should not be taken as evidence that no true effect exists. Although several theoretical mechanisms may propose that managing FA may contribute to eating difficulties through dietary restriction, anxiety, and hypervigilance, these results suggest that, in this sample, FA alone was not associated with a detectably higher likelihood of screening positive for an ED, and other psychological and environmental factors may play an important role.

An interesting finding was that FA status was found to be significantly associated with ED history. This suggests that there is some association between those with FA and having ever experienced an ED; however, this population may not be currently symptomatic. A further interesting finding was the significant association between FA status and having received ED treatment. If those with FA are more likely to have received treatment, this could further support an association between FA and a history of eating pathology that has since been treated; however, as this study is cross-sectional, current recovery status cannot be confirmed. This also may explain why those with FA did not show significantly higher rates of positive screening for an ED compared with those without FA. Both the SCOFF and EAT-8 questionnaires assess current ED behaviour and attitudes, which may have been resolved through treatment. There are multiple possible explanations as to why those with FA may have received treatment for an ED. These may include greater contact with healthcare professionals, leading to greater opportunity for identification and timely referral, and the possibility that disordered eating symptoms were misattributed to the FA itself, particularly in the absence of anaphylaxis, before being recognised and treated as an ED.

Duration of FA was retained in the multivariable model in preference to age on a priori theoretical grounds, reflecting cumulative exposure to dietary restriction and FA management rather than age itself. Within this model, shorter duration of FA was significantly associated with increased odds of positive screening for an ED. However, because duration and age were strongly correlated (r = 0.74) in this sample, and neither remained a significant predictor when entered together in a sensitivity analysis, this effect cannot be interpreted as reflecting a duration-specific mechanism independent of age; it may instead reflect a broader profile of younger or more recently diagnosed participants.

With this caveat in mind, several untested explanations for the pattern can be hypothesised. Those who have managed FA for longer may have recovered from, or received treatment for, an ED or may have developed greater confidence in managing their condition over time, reducing anxiety and disordered eating patterns; equally, adjustment to a new FA diagnosis and the accompanying sense of lost control common to chronic conditions [39] may contribute, given that restrictive food intake in anorexia nervosa has commonly been linked to feelings of control [40]. This pattern is consistent with the paediatric study whose ED classification was replicated here [33], which similarly found higher rates of disordered eating among children with a shorter FA duration. If replicated using designs able to separate duration from age, this association could have implications for early identification of comorbidity. For example, prompting greater clinical attention to emerging eating difficulties among those more recently diagnosed with FA, within primary care and from allergy physicians.

An alternative hypothesis draws on a conditioned fear model, where recently diagnosed individuals may still experience frequent or unpredictable reactions, with the resulting fear and hypervigilance manifesting as avoidant, ARFID-like restriction that lessens as safe foods become known and reactions grow less frequent. As this study was cross-sectional, causal or temporal conclusions cannot be drawn from these associations; longitudinal designs would be needed to establish whether FA duration precedes or follows changes in the likelihood of a positive ED screen.

Previous research shows the psychosocial impact and quality of life reduction in both ED and FA populations. Future research could investigate the psychosocial and quality of life experiences of those with co-occurring FA and ED. Comparative research examining how FA and ED contribute to psychosocial difficulties may help clarify areas of overlap between these conditions and inform more integrated approaches for assessment and intervention. It could also provide opportunity for greater collaboration between ED and FA clinicians.

Sex could not be entered into the multivariable model due to the low number of male participants who screened positive for an ED in this sample (n = 1). This finding is itself unsurprising and falls in line with previous research that females have a 3.5 times higher lifetime prevalence of EDs compared with males [41]. Equally, a history of ED was also associated with greater odds of screening positive for an ED in this sample, consistent with prior prospective research showing that ED history is one of the strongest predictors of future ED symptoms, with 26% of individuals with EDs known to relapse [42]. Together, ED history and duration of FA accounted for a modest proportion (approximately 14%) of the variance in positive ED screening status, suggesting that a substantial amount of variation remains unexplained by these factors alone and is likely attributable to the wider range of psychological and environmental influences discussed above.

The current study included a more diverse range of individuals compared with current census data when looking at ethnicity. However, the sample was heavily female-dominated (86%) compared with males (14%). Age was a further characteristic which was under-represented, which limits the generalisability of these findings across genders and the lifespan. A further limitation of the current study is that it used self-report measures. Although it was felt that anonymised questionnaires would be the more efficient method of assessing for possible EDs, there may be problems, including social desirability bias, inaccuracy of recall or reluctance to disclose sensitive information about current eating habits or mental health. Relatedly, our lifetime ED history item did not distinguish between current and resolved cases at the point of data collection, meaning its association with current positive screening may be partly definitional rather than purely reflecting a prospective or historical relationship. A recent systematic review found that studies using self-report measures to screen for an ED in those with FA typically had lower proportion screening positive compared with studies using other forms of assessment, which may further reflect why positive screening for an ED was not found to be significantly associated with FA here [29].

The gold standard for ED diagnosis is a semi-structured interview; as we were not diagnosing EDs and were replicating a previous study, the EAT-8 and SCOFF were selected instead as efficient, time-feasible screening measures suitable for a large, non-clinical, online sample [33,35,43]. However, neither measure has been validated in the FA population, for whom food restriction is often medically necessary: the SCOFF item “Does food dominate your life?”, for example, may apply to someone with FA without an ED simply due to label-checking or allergen avoidance. It is important to note that the use of EAT-8 and SCOFF within FA populations raises questions regarding construct validity. Some items may capture behaviours characteristically found in ED but also may occur due to reasons relating to FA management (e.g., food avoidance). Therefore, it is important to consider that elevated scores on the screening measures may reflect limitations in the ability of these instruments to distinguish between allergy-related food avoidance and restriction, and ED behaviour. The SCOFF’s moderate specificity suggests that positive screens should be interpreted cautiously in this population, and this measurement limitation may partly explain the regression model’s weaker performance at identifying true positives, correctly classifying fewer than half of those who actually screened positive. Moreover, the SCOFF’s comparatively low internal consistency in this sample (α = 0.54–0.62) may reduce its reliability as a screening tool and could contribute to measurement error affecting both the prevalence estimates and the regression results.

Notably, over a third of participants in both groups screened positive overall, a rate considerably higher than typical general population estimates [44]. One possible contributor is selection effects: because the survey was explicitly framed around food, eating, and allergy management, individuals with pre-existing eating or body-image concerns may have been more inclined to participate than in a general population sample, potentially inflating the observed rate independent of any measurement issue. This apparent inconsistency with the self-report under-detection discussed above likely reflects a difference in what is being compared. Self-report typically underestimates prevalence relative to clinical interviews, the gold standard. In contrast, the elevated rate observed here relative to general population studies more plausibly reflects the limited specificity of these screening tools in an FA population, alongside possible selection effects, rather than a genuine over-detection of EDs overall. While this may reflect genuine rate within this sample, it could also indicate over-identification by the screening tools, given their imperfect specificity, and warrants caution when interpreting the figures reported here. Both measures were also designed primarily to identify EDs characterised by weight and shape concerns, and so may fail to capture conditions such as ARFID. This is illustrated by a recent mixed-methods study of children with FA, which found a low rate of positive screening for ARFID-related symptoms using an ARFID-specific tool (4.3%), despite the majority of the same sample showing moderate to high food neophobia and pronounced selective eating. This highlight how substantially estimates can diverge, depending on whether a general or ARFID-specific instrument is used [45].

## 5. Conclusions

Overall, no statistically significant association between FA status and positive ED screening was detected in this sample, including after adjusting for age and sex, suggesting FA alone was not associated with a higher likelihood of screening positive for an ED in this sample. However, FA was associated with a lifetime history of ED and ED treatment, suggesting this relationship may be more evident earlier on, or among those who have since recovered. Among those with FA, positive screening was significantly associated with ED history and shorter FA duration in a multivariable model, together explaining only a modest proportion of variance and pointing to a broader range of contributing factors; however, because FA duration and age were highly correlated, the duration effect could not be shown to be independent of age, and may instead reflect a broader profile of younger or more recently diagnosed participants. Sex was significantly associated with positive screening at the bivariate level, though this could not be assessed in a multivariable model due to the small number of male participants who screened positive. Given the use of screening measures not validated in the FA population and a sample skewed toward young, female participants, these findings should be considered to be preliminary. Future research using longitudinal designs, FA-validated tools, and more diverse samples would help clarify when and how the FA experience relates to disordered eating. Longitudinal research could examine the relationship between FA and ED symptoms across the transition from adolescence to young adulthood. This may be particularly important given that FA can resolve during childhood, persist into adulthood, or emerge later in life, whereas adolescence appears to represent a key developmental period for EDs’ onset, with peak and median ages of onset estimated at 15.5 and 18 years, respectively [46].

## Figures and Tables

**Table 1:** Demographic and clinical information.

Characteristic	No FA (n = 237)	FA (n = 134)
*Sex at birth, n (%)*		
Female	203 (85.7)	116 (86.8)
Male	34 (14.3)	18 (13.4)
*Described gender, n (%)*		
Woman	198 (83.5)	115 (85.8)
Man	34 (14.3)	18 (13.4)
Non-binary	5 (2.1)	1 (0.7)
*Age, mean years (SD)*	24.57 (11.79)	29.22 (11.87)
*Ethnicity, n (%)*		
Asian or Asian British	38 (16.0)	13 (9.7)
Black, Black British, Caribbean, or African	5 (2.1)	3 (2.2)
Mixed or multiple ethnic groups	11 (4.6)	12 (9.0)
Other ethnic group	4 (1.7)	1 (0.7)
White	179 (75.5)	105 (78.4)
*ED history, n (%)*		
Ever had an ED	49 (20.7)	48 (36.4)

Standard deviation (SD). Percentages are calculated based on valid responses. Data on ED history were missing for 2 participants in the FA group (valid n = 132).

**Table 2:** Bivariate correlations among positive screening for ED and candidate predictor variables.

Variable	1	2	3	4	5	6	7	8
1. Positive screening for ED								
2. Length of FA (years)	−0.21 *							
3. Treatment for ED	0.16	−0.17 *						
4. ED history	0.29 ***	−0.06	0.62 ***					
5. Sex	0.26 **	−0.08	0.18 *	0.16				
6. Adrenaline autoinjector prescription	−0.07	0.29 ***	−0.03	−0.05	−0.04			
7. Number of allergens	0.07	0.38 ***	−0.01	0.02	0.03	0.46 ***		
8. Ethnicity	0.03	0.13	0.01	−0.02	0.06	0.07	0.12	
9. Age	−0.19 *	0.74 ***	−0.12	−0.06	−0.03	0.08	0.15	0.19 *

N = 130–134 (2 cases missing on ED history, 4 on length of FA), handled via pairwise deletion. * *p* < 0.05, ** *p* < 0.01, *** *p* < 0.001. Ethnicity was coded to a binary of White vs. non-White due to small cell-counts.

**Table 3:** Logistic regression predicting positive screening for ED.

Variable	B	SE	Wald	*df*	*p*	OR	95% CIs
ED history (positive)	1.12	0.39	8.17	1	0.004	3.07	[1.42, 6.61]
Length of FA (years)	−0.04	0.02	4.39	1	0.036	0.97	[0.94, 0.998]
Constant	−0.28	0.39	0.54	1	0.464	0.76	—

N = 128 (6 cases excluded: 2 missing ED history, 4 missing length of FA), handled via listwise deletion. Nagelkerke R^2^ = 0.140. Model χ^2^(2) = 13.87, *p* < 0.001. Beta (B); standard error (SE); degrees of freedom (*df*).

## Data Availability

The data presented in this study are available on reasonable request from the corresponding author due to restrictions arising from the terms of participant consent, which did not provide for data sharing beyond the research team.

## Abbreviations

The following abbreviations are used in this manuscript.

FA	Food allergy
ED(s)	Eating disorder(s)
ARFID	Avoidant/restrictive food intake disorder
SD	Standard deviation
CI	Confidence interval
OR	Odds ratio
SE	Standard error
*df*	Degrees of freedom
B	Beta-unstandardised regression coefficient
